# Expansion of Endothelial Progenitor Cells in High Density Dot Culture of Rat Bone Marrow Cells

**DOI:** 10.1371/journal.pone.0107127

**Published:** 2014-09-25

**Authors:** Yang Lu, Yiyi Gong, Jie Lian, Ling Wang, James D. Kretlow, Guangdong Zhou, Yilin Cao, Wei Liu, Wen Jie Zhang

**Affiliations:** 1 Department of Plastic and Reconstructive Surgery, Shanghai 9^th^ People’s Hospital, Shanghai Jiao Tong University School of Medicine, Shanghai Key Laboratory of Tissue Engineering, National Tissue Engineering Center of China, Shanghai, China; 2 Division of Plastic Surgery, Baylor College of Medicine, Houston, Texas, United States of America; Cardiological Center Monzino, Italy

## Abstract

In vitro expansion of endothelial progenitor cells (EPCs) remains a challenge in stem cell research and its application. We hypothesize that high density culture is able to expand EPCs from bone marrow by mimicking cell-cell interactions of the bone marrow niche. To test the hypothesis, rat bone marrow cells were either cultured in high density (2×10^5^ cells/cm^2^) by seeding total 9×10^5^ cells into six high density dots or cultured in regular density (1.6×10^4^ cells/cm^2^) with the same total number of cells. Flow cytometric analyses of the cells cultured for 15 days showed that high density cells exhibited smaller cell size and higher levels of marker expression related to EPCs when compared to regular density cultured cells. Functionally, these cells exhibited strong angiogenic potentials with better tubal formation in vitro and potent rescue of mouse ischemic limbs in vivo with their integration into neo-capillary structure. Global gene chip and ELISA analyses revealed up-regulated gene expression of adhesion molecules and enhanced protein release of pro-angiogenic growth factors in high density cultured cells. In summary, high density cell culture promotes expansion of bone marrow contained EPCs that are able to enhance tissue angiogenesis via paracrine growth factors and direct differentiation into endothelial cells.

## Introduction

Stem cell based therapy for ischemic diseases of the cardiovascular system has become an important area of stem cell research and translation. Endothelial progenitor cells (EPCs), which were first discovered in circulating blood [1], have been intensively investigated for their ability to enhance tissue angiogenesis and attenuate ischemic injury in both animal models and patients [2]. To achieve the desired therapeutic effect, a large amount of EPCs are normally required for a single injection, which presents a great challenge due to the extremely low number of EPCs in both circulating blood and bone marrow [3]. Thus, efficient expansion of EPCs in culture becomes a prerequisite for their therapeutic application. Many attempts have been made to expand EPCs in culture, including the pre-coating of culture dishes with extracellular matrix (ECM) proteins and the addition of growth factors to the culture medium [4], [5]. Additionally, high costs and safety concerns when using growth factors hinder the clinical application of EPC-based therapy. Therefore, the establishment of an ideal culture method to expand EPCs without the need for growth factors is a critical goal to facilitate clinical translation.

The stem cell niche is a well known microenvironment regulating self-renewal of stem cells in the body [6], [7]. The key components of the niche include growth factors and ECM secreted by surrounding cells, cell-cell interactions, as well as other biochemical and biophysical factors [8], [9]. Therefore, it will be ideal to mimic this niche during in vitro expansion of stem cells [10], [11]. Despite the broad application of ECM pre-coating and the addition of growth factors for EPC expansion, mimicking cell-cell interaction is usually neglected due to the low cell-seeding density in these studies [12]. We hypothesized that high density cell culture of bone marrow cells might be able to enrich contained EPCs during in vitro expansion via better mimicking cell-cell interactions present in the stem cell niche.

To test this hypothesis, rat bone marrow cells were cultured at high density in dots and compared with those cultured at regular density. Expanded cells were characterized with flow cytometric analyses, and their angiogenic potentials were evaluated in vitro with capillary tube formation assay and in vivo with an ischemic hind limb rescue model. Global gene expression profiles were also compared with gene-chip analysis to reveal the key differences between cells expanded in high and low densities.

## Materials and Methods

### 1. Experimental animals

Male Wistar rats (4-weeks-old) and nude mice (6-weeks-old) were purchased from Shanghai Chuansha Experimental Animal Raising Farm (Shanghai, China). Animal study protocols were approved by The Animal Care and Experiment Committee of Shanghai Jiao Tong University School of Medicine.

### 2. Isolation and primary culture of bone marrow cells

Rat bone marrow cells were extracted from the femurs of 4-week-old male Wistar rats. To remove the majority of the non-adherent blood cells, primary culture of bone marrow cells was performed by seeding the cells at 1.6×10^4^ cells/cm^2^ in Dulbecco’s modified Eagle’s medium (DMEM; Invitrogen, Carlsbad, CA, USA) with 10% fetal bovine serum (FBS; HyClone, Logan, UT, USA) and 0.2% penicillin/streptomycin (Sigma, St. Louis, MO, USA). Medium was changed every 3 days. After 6–7 days of culture, the primary adherent cells (P0) were harvested with using trypsin/EDTA (0.25% w/v trypsin, and 0.02% EDTA; Invitrogen), and then subcultured at a different density.

### 3. Cell culture at regular or high density

For regular density culture, 9×10^5^ primary cultured cells were seeded evenly at a density of 1.6×10^4^ cells/cm^2^ in a 10-cm diameter tissue culture dish in 10 ml DMEM with 10% FBS. Cells were passaged in the same manner every 3 days.

For high density culture, a dot culture system was established. Briefly, 9×10^5^ primary cells (equal to the cell number of regular density culture) were suspended in 300 µl of culture medium, and then six drops (50 µl each) of cell suspension were dot-seeded separately onto a 10-cm diameter culture dish with equal distance. The average diameter of each dot was 1 cm, resulting in a final local cell density of 2×10^5^ cells/cm^2^. The culture dish was placed in an incubator for 30 min, and then culture medium in 10 ml was gently added to cover the dish. Medium was changed every 3 days, and the cells were passaged in the same manner every 6 days.

### 4. DiI-Ac-LDL-lectin staining

Cells were incubated with 2.4 µg/ml 1,1′-dioctadecyl-3,3,3′,3′-tetra-methylindocarbocyanine-labeled acetylated low density lipoprotein (DiI-ac-LDL; Invitrogen) at 37°C for 4 h, followed by fixation with 4% paraformaldehyde for 20 min. After washing with phosphate buffered saline (PBS), the cells were counterstained with fluorescein isothiocyanate (FITC)-conjugated lectin from *Ulex europaeus* (UEA; Sigma).

### 5. Flow cytometric analyses

After trypsinization, aliquots of 2×10^5^ cells suspended in 200 µl washing buffer (PBS containing 2% FBS) were incubated on ice for 30 min with phycoerythrin (PE)- or FITC-conjugated antibodies. PE- and FITC-conjugated isotype matching immunoglobulins were used as a control. After staining and washing, cells were analyzed on a flow cytometer (Epics Altra; Beckman Coulter, Fullerton, CA, USA). Antibodies against the following markers were used: CD29, CD45, CD90, CD31 (BD Biosciences, San Diego, CA, USA), CD14, CD34, CD144 (Santa Cruz Biotechnology, Santa Cruz, CA, USA), KDR, and CD133 (Abcam, Cambridge, UK). Flow cytometric data were analyzed with CXP software (Beckman Coulter).

### 6. Cell sorting, endothelial differentiation and immunofluorescence staining

Cells derived from high density culture at day 15 were stained with a biotinylated anti-rat-CD45 monoclonal antibody (BD Biosciences) for 30 min on ice, washed, and then incubated with streptavidin-conjugated magnetic beads (Miltenyi Biotec, Teterow, Germany) for 20 min. Cells were then separated into CD45^+^ and CD45^−^ cells using magnetic columns (Miltenyi Biotec) and plated onto fibronectin-coated chamber slides (BD Biosciences) in EGM-2 medium supplemented with 50 ng/ml vascular endothelial cell growth factor (VEGF, Lonza, Walkersville, MD, USA). After 14 days of induction, cells were incubated with DiI-ac-LDL (Invitrogen) as previously described, or fixed and stained with anti-von Willebrand factor (vWF) (Santa Cruz Biotechnology) or anti-endothelial nitric oxide synthase (eNOs) (BD Biosciences) antibodies and visualized with an Alexa Fluor 488-conjugated secondary antibody. Three representative fields were recorded and the positive cells were calculated by Image-Pro Plus software (Media Cybernetics, Rockville, MD, USA).

### 7. In vitro angiogenesis assay

Matrigel (BD Biosciences) basement membrane matrix was added to 24-well plates and incubated for 30 min at 37°C to allow gel solidification. Then, 2×10^4^ cells in 500 µl EGM-2 were seeded onto the gel. Twelve hours later, the plates were observed under a microscope. Nine representative fields were recorded and the average number of branch points was calculated by Image-Pro Plus software (Media Cybernetics).

### 8. Murine model of hind limb ischemia, rescue with cell transplantation, and laser Doppler evaluation

The hind limb ischemia model was made in 6-week-old male athymic nude mice. Briefly, the left femoral artery was ligated and excised below the inguinal ligament and above the bifurcation of the popliteal artery. Twenty-four hours later, mice were intramuscularly injected with 1×10^6^ 1,1′-dioctadecyl-3,3,3′,3′-tetra-methylindocarbocyanine dye (CM-DiI; Invitrogen)-labeled cells in 100 µl PBS into the ischemic limbs. Cells from high density and regular density culture at day 15 were injected respectively. Mice that received the same volume of PBS served as a control. Ten animals were injected in each group. Blood perfusion of the hind limbs was detected by a laser Doppler perfusion imager (Moor Instruments, Devon, UK) on days 1 and 21 post-treatment. The recovery of perfusion was calculated as the ratio of ischemic to non-ischemic hind limb blood perfusion.

### 9. Histological analyses

Three weeks after cell transplantation, the mice were sacrificed and the adductor muscles were harvested from the ischemic limbs. Samples were embedded in OCT compound, snap-frozen in liquid nitrogen, and cut into 10 µm-thick sections. Three sections from each mouse were stained with FITC-conjugated isolectin B4 (Sigma) and DAPI (Invitrogen), observed under a confocal microscope (Leica, Wetzlar, Germany). Four fields from each tissue section were randomly selected, and the number of capillaries was counted. Co-localization of CM-DiI-labeled cells with isolectin B4-labeled endothelial cells was also observed. Three representative fields were recorded and the percentages of donor-derived endothelial, non-endothelial cells and that of recipient-derived endothelial cells were calculated by Image-Pro Plus software (Media Cybernetics).

### 10. Microarray analysis

After 15 days of culture at regular or high density, total RNA was extracted from the cells with Trizol (Invitrogen). The Rat 4×44 K Gene Expression Array was used in this study (Agilent Technology, Santa Clara, CA, USA). Sample labeling and array hybridization were performed according to the Agilent One-Color Microarray-Based Gene Expression Analysis protocol. Agilent Feature Extraction software (version 11.0.1.1) was used to analyze the acquired array images. Differentially expressed genes were identified by fold change filtering. Hierarchical clustering and pathway analysis were performed using Agilent GeneSpring GX software (version 12.0). Experiments were repeated with three pairs of cultured cell samples.

### 11. qRT-PCR

Total RNA extracted from day 15 cultured cells, sorted CD45^+^ and CD45^−^ cells were reverse transcribed into cDNA and subsequently amplified using a Power SYBR Green PCR master mix (2×) (Applied Biosystems, Foster City, CA, USA) in a real-time thermal cycler (Mx3000PTM QPCR System; Stratagene, La Jolla, CA, USA). Primers are listed in Table S1. qRT-PCR was conducted in triplicate for each sample. Gene expression was normalized to glyceraldehyde-3-phosphate dehydrogenase (GAPDH) expression. Experiments were repeated with three pairs of cultured cell samples.

### 12. Analysis of growth factors in culture supernatants

Supernatants from day 3 cell cultures were collected and growth factors in the supernatants, including VEGF, platelet-derived growth factor (PDGF), transforming growth factor-β (TGF-β), hepatocyte growth factor (HGF), basic-fibroblast growth factor (bFGF) and stromal cell-derived factor-1 (SDF-1), were measured by ELISA kits (R&D Systems, Minneapolis, MN, USA) following the manufacturer’s instructions. To detect the angiogenic inductive potential of the supernatants, 2×10^4^ human umbilical vein endothelial cells (HUVECs; Sciencell, Carlsbad, CA, USA) were seeded onto a Matrigel-coated 24-well plate in 500 µl of supernatants from regular or high density cultures. Cells incubated with 500 µl DMEM with 10% FBS served as a control. Twelve hours later, the cells were observed under a microscope and the total number of branch points was counted by Image-Pro Plus software. Experiments were repeated with three pairs of cultured cell samples.

### 13. Statistical analysis

Data were expressed as the mean ± standard deviation. Comparisons between groups were analyzed by Students t-test or ANOVA for experiments with more than 2 subgroups. A value of p<0.05 was considered statistically significant.

## Results

### 1. Endothelial progenitor-like cells in high density culture

**Figure 1:**
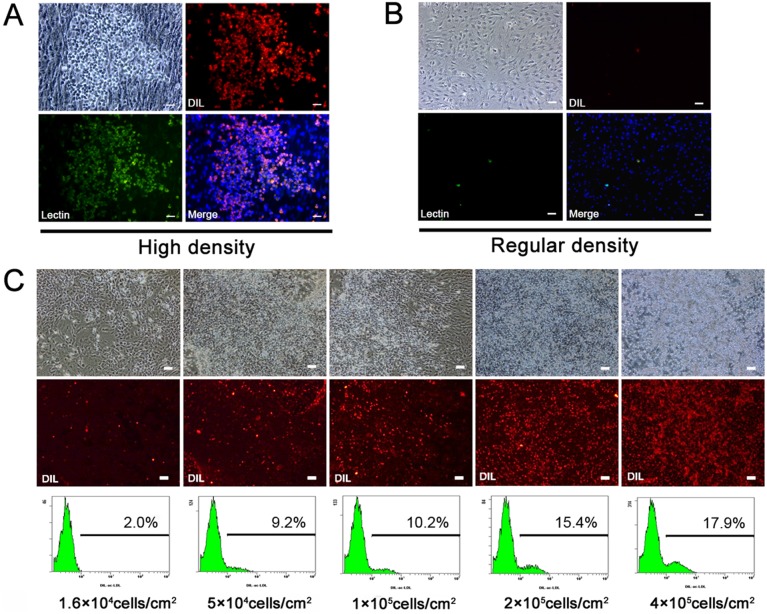
Bone marrow cells in high density culture. A, Small bright cells were observed in high density culture (2×10^5^ cells/cm^2^) of rat bone marrow cells. Cells were incubated with DiI-ac-LDL and stained with FITC-conjugated UEA lectin and DAPI. Small bright cells were double-positive for DiI-ac-LDL and UEA lectin with counterstained DAPI. B, Spindle-shaped cells were observed in regular density culture (1.6×10^4^ cells/cm^2^). The majority of cells were negative for DiI-ac-LDL uptake and UEA lectin binding. C, Bone marrow cells were seeded at different densities. After 3 days of culture, fluorescence microscopic observation and flow cytometric analysis revealed an increase of DiI-ac-LDL-positive cells with the increase of cell seeding density (n = 3). Scale bars, 100 µm.

Cell seeding density could determine cell biological behavior and function in vitro [13], [14]. To test its effect on bone marrow EPC survival and proliferation, rat bone marrow cells were first cultured in tissue culture dishes for 7 days to remove the majority of the non-adherent blood cells. Adherent cells from primary culture (P0) were then collected and seeded at various densities from a regular density of 1.6×10^4^ cells/cm^2^ to a relatively high density of 4×10^5^ cells/cm^2^ in tissue culture dishes. Surprisingly, a population of small bright cells was observed in the high density culture after 3 days of incubation. The small bright cells were able to uptake DiI-ac-LDL and bind to UEA lectin (Fig. 1A), suggesting that they were likely EPCs.[4] In contrast, few small bright cells were observed in the regular density culture (Fig. 1B). Flow cytometric analysis confirmed that with increased seeding density, the percentage of DiI-ac-LDL-positive cells increased from 2.0% to 17.9% (Fig. 1C). A regular density of 1.6×10^4^ cells/cm^2^ and an optimal high density of 2×10^5^ cells/cm^2^ were then chosen to test the effects of seeding density on cell behavior in the following experiments.

### 2. High density cultured cells express higher levels of EPC markers

**Figure 2:**
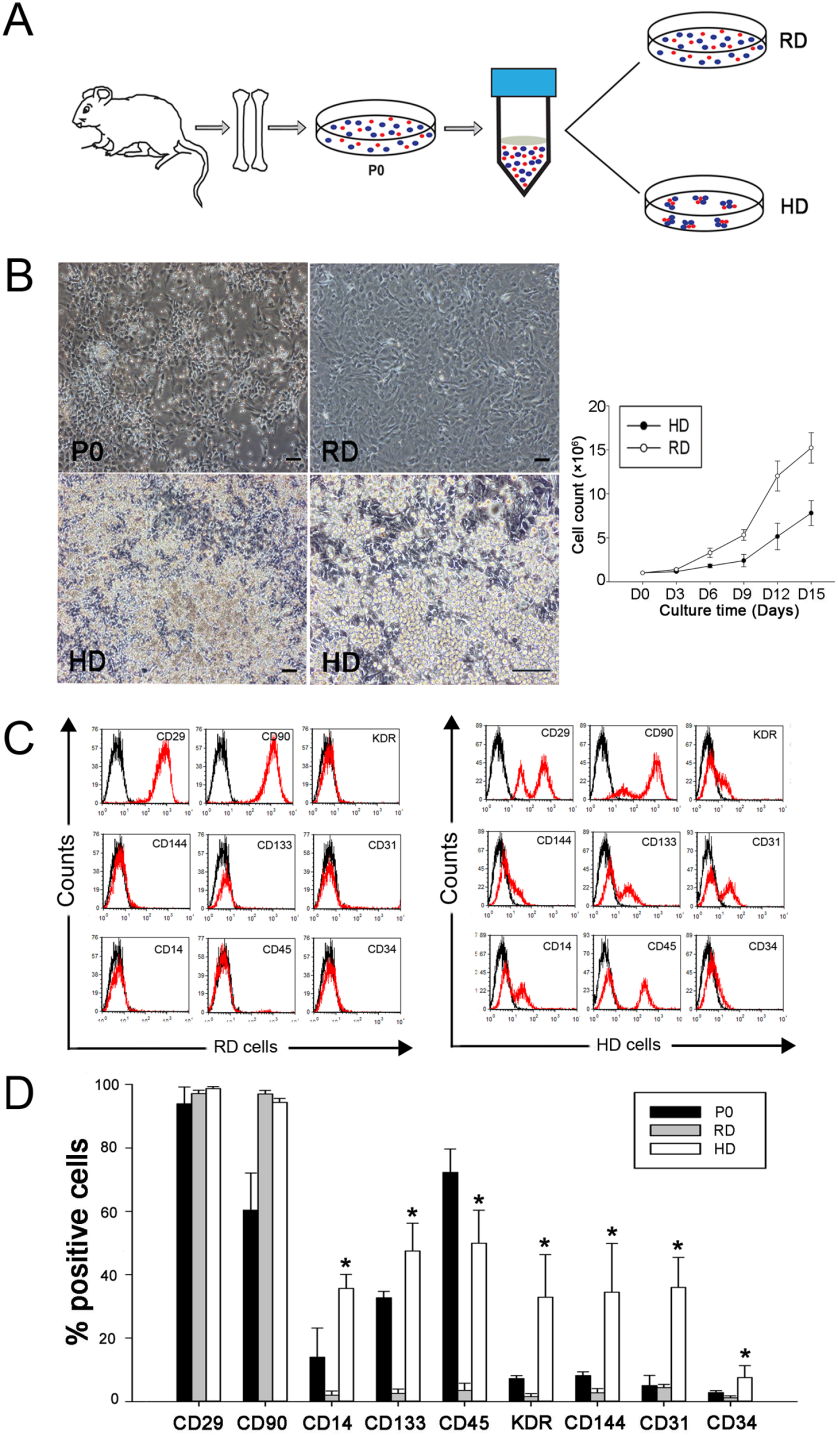
Characterization of bone marrow cells cultured in high density dots. A, Diagram of the high density dot culture system. Adherent cells from primary cultures (P0) of rat bone marrow aspirates were subcultured at a regular density (RD) or in high density dots (HD). B, Typical morphologies of primary cells (P0), the cells in RD- and HD-culture at day 15, and the proliferation of cells in RD- and HD-culture (n = 3). Scale bars, 100 µm. C, Representative histograms of cell surface marker expression analyzed by flow cytometer. D, Quantitative analysis of the flow cytometric data showed higher expression levels of EPC related markers in high density cultured cells (n = 3). *p<0.05.

When an entire 10-cm diameter culture dish was seeded with cells at a density of 2×10^5^ cells/cm^2^ (1.12×10^7^ cells/dish), the nutrients in the medium were exhausted within a day (data not shown). To avoid the rapid exhaustion of nutrients in high density culture, we established a dot culture system. Briefly, 9×10^5^ cells were seeded into six high density dots at a local density of 2×10^5^ cells/cm^2^ in a 10-cm diameter culture dish. As a control, the same number of cells was seeded evenly in a 10-cm diameter culture dish, resulting in a regular density of 1.6×10^4^ cells/cm^2^ (Fig. 2A). After 15 days of culture, significantly more small bright cells were observed in high density culture than in regular density culture (Fig. 2B). The small bright cells grew on top of the spindle-shaped cells. Cell counting showed that the cells in high density culture had expanded about 6 times (2.5 cell doublings) in 15 days and the cells in regular density culture had expanded about 16 times (4 cell doublings) (Fig. 2B).

Flow cytometric analyses showed that primary cells (P0) expressed high levels of CD29 (92%), CD 90 (60%), CD45 (73%) and CD133 (32%) but low levels (<15%) of CD14, KDR, CD144, CD31 and CD34 (Fig. 2C, 2D). Interestingly, compared to P0 cells, cells in high density culture expressed higher levels of CD14, CD133, KDR, CD144, CD31 and CD34, which are known makers for EPCs. However, those markers were significantly decreased in regular density cultured cells. In addition, CD45 expression was significantly decreased in the regular density cultured cells but maintained in the high density cultured cells. These results suggest that cells derived from high density culture might contain more EPCs.

### 3. EPCs are enriched in the CD45^+^ cell population

**Figure 3:**
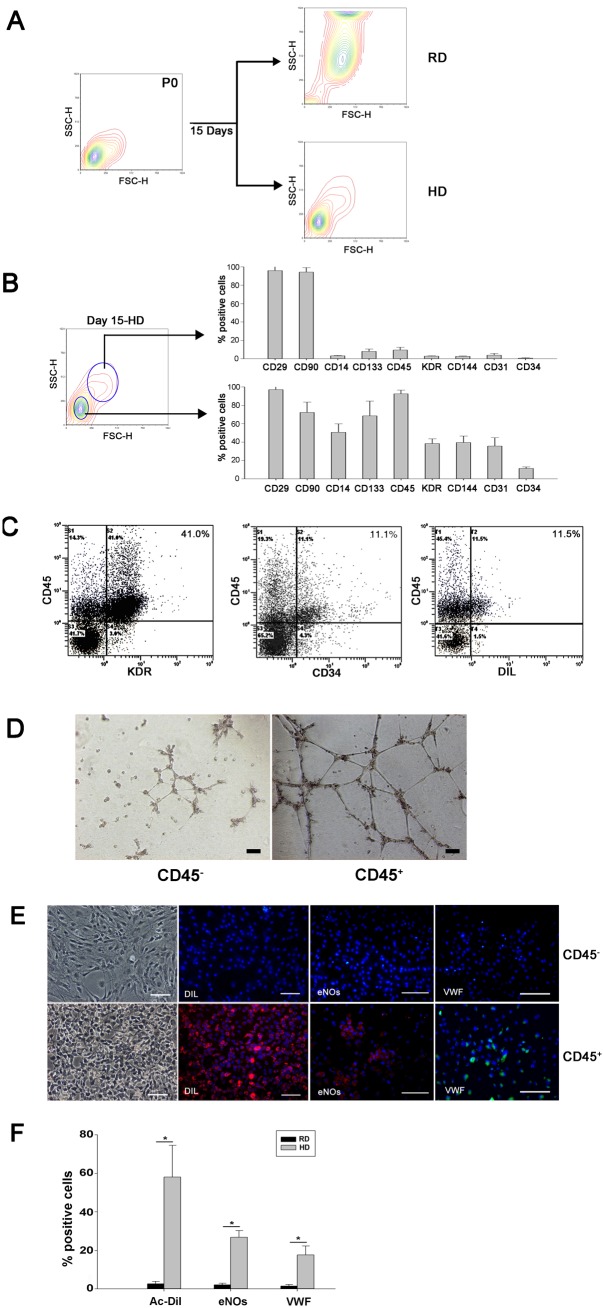
Endothelial precursors are enriched in the CD45^+^ cell population. A, Flow cytometric analysis showed that the majority of cells in regular density (RD) culture became larger after their expansion, whereas the cells in high density (HD) culture maintained the small size of their parental cells (P0). B, Cells from high density culture were analyzed further by gating based on different cell sizes. The small cells showed higher expression levels of EPC related markers than those of the large cells. C, In high density culture, the majority of cells positive for KDR, CD34 and DiI were also positive for CD45. D, In vitro angiogenesis assay of CD45^+^ and CD45^−^ cells sorted from high density culture. E, CD45^+^, but not CD45^−^, cells from high density culture differentiated into endothelial cells that were able to uptake DiI-ac-LDL and express vWF and eNOs. Scale bars, 100 µm. F, Percentages of endothelial cell marker-positive cells in CD45^+^ and CD45^−^ populations after 14 days of induction (n = 3). *p<0.05.

Forward/side scatter analysis from flow cytometric data showed that the majority of cells in high density culture maintained their small size, similar to that of their parent cells (P0), whereas the majority of cells in regular density culture became larger after culture (Fig. 3A). The relationship between cell size and surface marker expression was analyzed by gating on the small and large cells separately. In high density culture, the small cells expressed higher levels of CD14, CD133, CD45, KDR, CD144, CD31 and CD34 than large cells (Fig. 3B), indicating that cell surface marker expression level was closely related to the size of cells.

Since the majority (90%) of small cells in high density culture expressed CD45 (Fig. 3B), we speculated that KDR^+^, CD144^+^, CD31^+^ and CD34^+^ cells were likely within the CD45^+^ cell population. Indeed, dual color analysis by flow cytometer confirmed that the majority of KDR^+^, CD34^+^ or DiI-ac-LDL^+^ cells co-expressed CD45 (Fig. 3C). Cells from high density culture at day 15 were then sorted into CD45^+^ and CD45^−^ populations. In vitro angiogenesis assay showed that more tube-like structures were formed in CD45+ population (Fig. 3D). After 14 days of subculture in the presence of VEGF, immunofluorescence staining revealed that cells from the CD45^+^, but not CD45^−^, population were able to uptake DiI-ac-LDL and expressed eNOs and vWF, (Fig. 3E, F), suggesting that the majority of EPCs were within the CD45^+^ population.

### 4. High density cultured cells have a higher angiogenic potential

**Figure 4:**
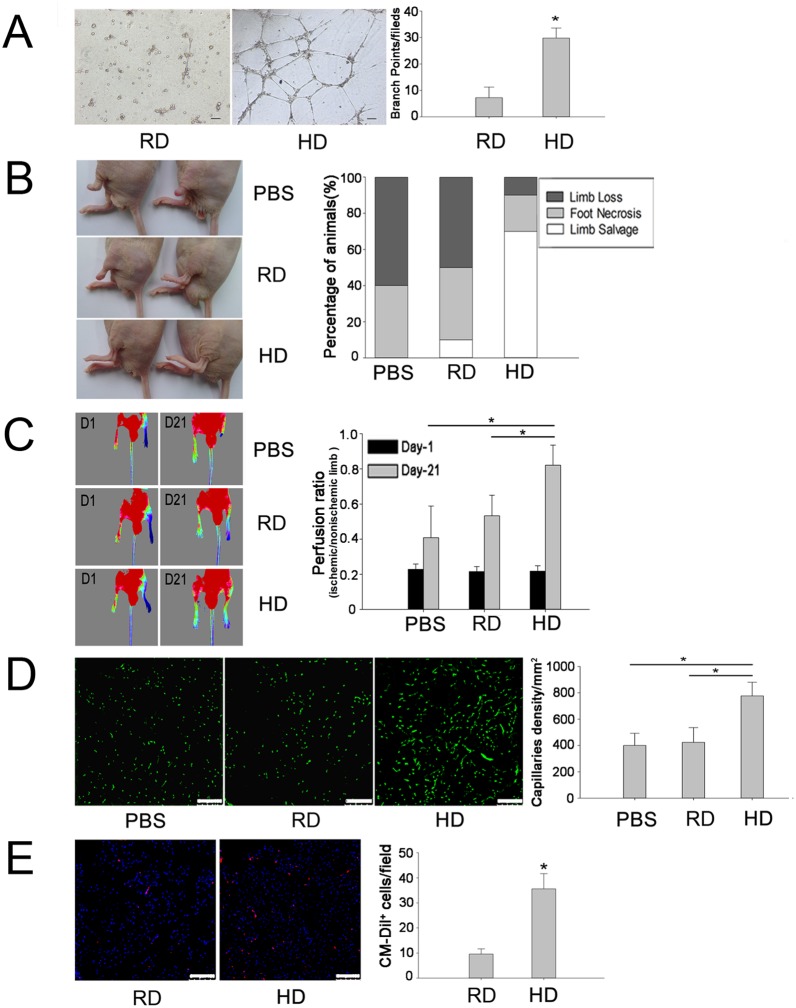
Pro-angiogenic potential of high density cultured cells in vitro and in vivo. A, In vitro tube formation assay with Matrigel for the cells derived from high density (HD) and regular density (RD) groups with quantification of the number of branch points per field (n = 9). *p<0.05, Scale bars, 100 µm. B, An in vivo angiogenic assay was performed by transplantation of expanded cells into ischemic hind limbs of nude mice. Representative views of ischemic left hind limbs at 3 weeks after treatment with PBS, regular density cultured cells or high density cultured cells and percent distribution of outcomes after treatment (n = 10). C, Laser Doppler images of blood perfusion with mice in a supine position at day 1 after femoral artery ligation and at day 21 after cell transplantation or PBS injection. Restoration of blood flow was calculated by comparing the laser Doppler image data of ischemic left limbs with that of non-ischemic right limbs at day 21. A significant improvement of blood supply was achieved in the group treated with high density cultured cells (n = 4). *p<0.05. D, Blood vessels in hind limb adductor muscles were identified by ILB4 staining. A higher number of capillaries were observed in the group treated with high density cultured cells (n = 4). *p<0.05, Scale bars, 100 µm. E, Donor-derived cells were detected by CM-DiI labeling of hind limb adductor muscles. A higher number of CM-DiI^+^ cells were observed in the muscles treated with high density cultured cells (n = 5). *p<0.05, Scale bars, 100 µm.

To test the angiogenic potential of expanded cells in vitro, the tube formation assay of cells after 15 days expansion was performed. As shown in Figure 4A, high density cultured cells formed an obvious tubular network whereas no connected vessel tube was observed in the regular cultured cell group. The number of branch points per field was significantly higher in the high density group than in the regular density group (Fig. 4A), supporting the fact that high density culture enriched EPCs.

The angiogenic potential of expanded cells was further tested in vivo in an ischemic hind limb model. At day 21 post-transplantation, injection of high density cultured cells achieved limb salvage in 7 out of 10 mice, whereas only 1 out of 10 mice were fully protected in the regular density group and no limb salvage was achieved in the PBS-treated group (Fig. 4B). Laser Doppler image analysis revealed that blood flow was restored to 80% in the high density group, whereas blood flow was restored to 55% and 40% in regular density and PBS groups, respectively (Fig. 4C). Histological analysis confirmed that the number of microvessels in ischemic muscles was significantly higher in the high density group than that in the regular density or PBS groups (Fig. 4D). In addition, more CM-DiI-labeled cells were observed in the high density group than in the regular density group (Fig. 4E).

### 5. Integration of donor cells in neovascularization

**Figure 5:**
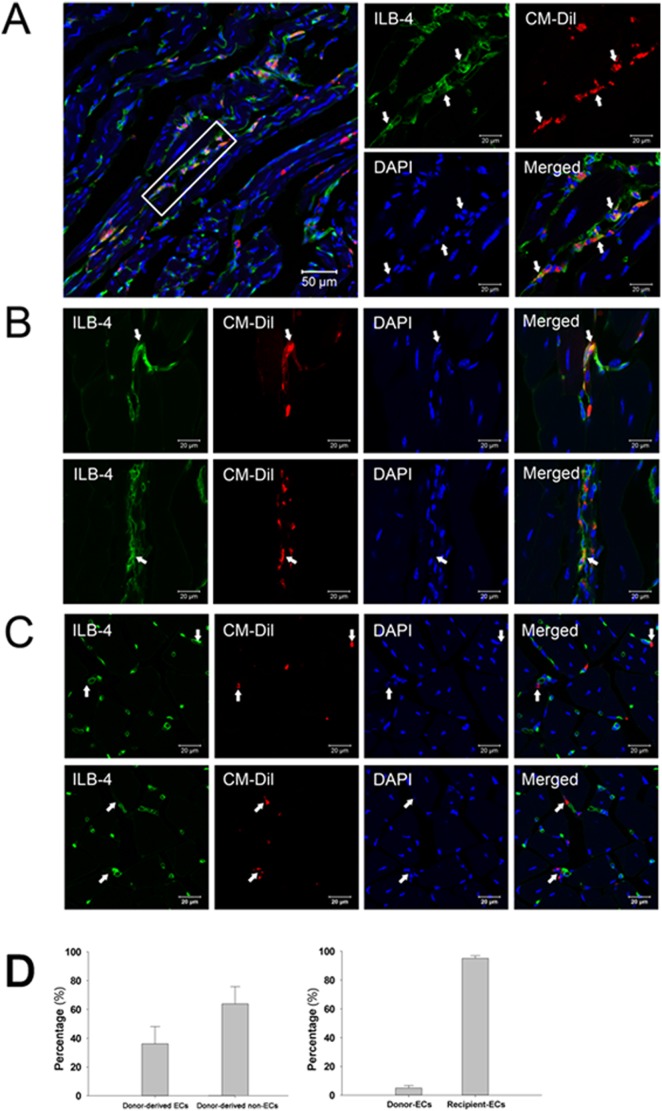
Engraftment and endothelial differentiation of high density cultured cells in ischemic limbs. A and B, Confocal images revealed that some of high density cultured cells (CM-DiI^+^, white arrows) participated in neovascularization by direct differentiation into endothelial cells (ILB4^+^). C, CM-DiI^+^ cells (white arrows) close to capillaries, but negative for ILB4 staining, might support vessel formation via paracrine effect of released pro-angiogenic growth factors. D, The percentages of endothelial and non-endothelial differentiation of injected cells, and the percentage of vessels derived from donor and recipient (n = 3). *p<0.05.

To further evaluate the role of high density cultured cells in enhanced neovascularization, tissue sections were stained with isolectin-B4 and observed under a confocal microscope. As shown in Figure 5A and 5B, co-localization of CM-DiI and isolectin-B4 was observed in the neo-capillaries, indicating that some of the injected cells had differentiated into endothelial cells and incorporated into the vessel wall. However, some CM-DiI-labeled cells were close to the capillaries but negative for isolectin-B4 staining (Fig. 5C), suggesting that these cells might support vessel formation in an indirect manner. Statistic analyses by Image-Pro Plus software showed that about 36.1% of CM-DiI-labeled cells differentiated into endothelial cells (co-stained with isolectin-B4), and about 4.9% of vessels were derived from injected cells (Fig. 5D).

### 6. Up-regulation of cell adhesion molecules and pro-angiogenic growth factors in high density cultured cells

**Figure 6:**
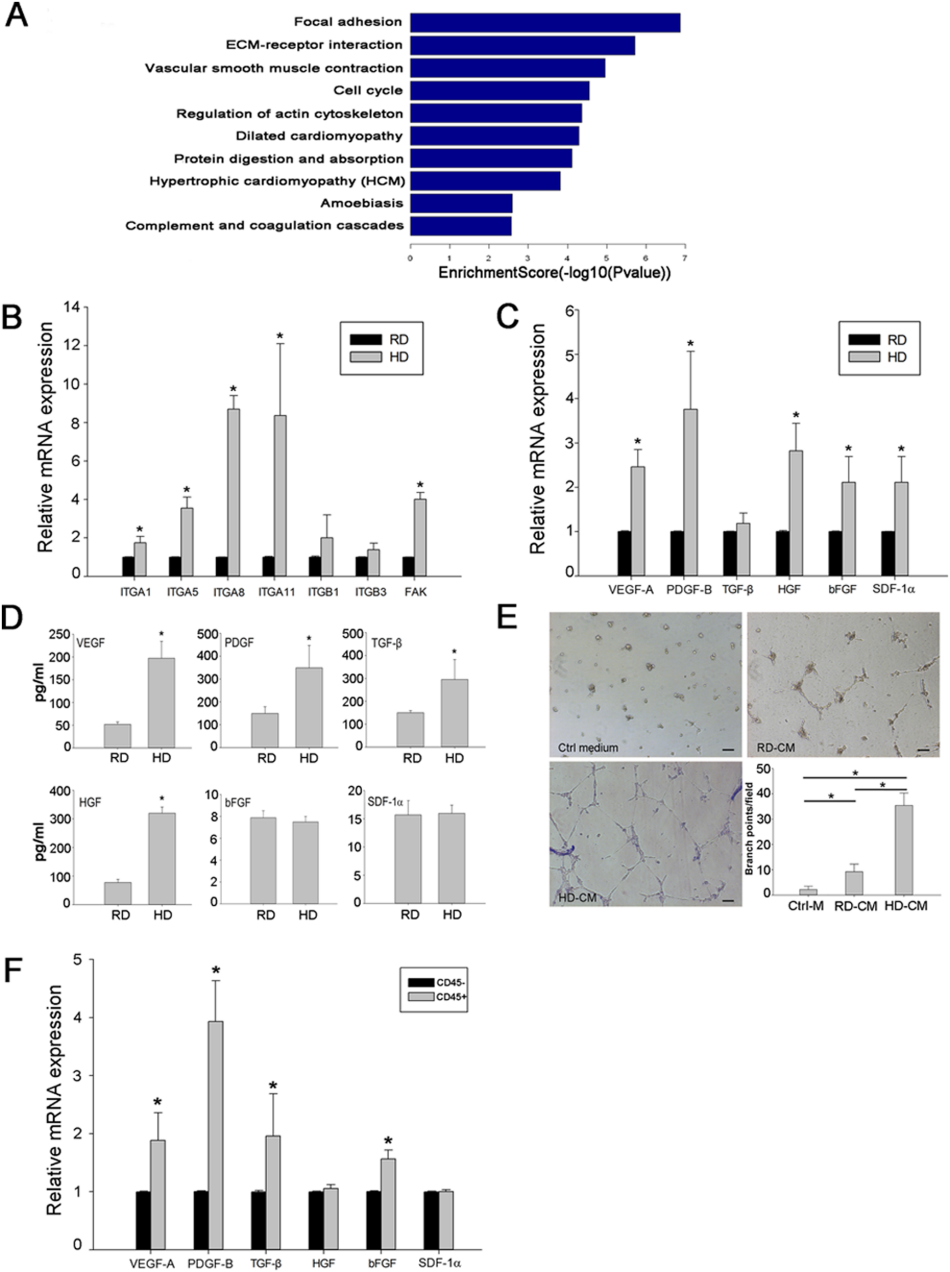
Up-regulation of cell adhesion molecules and pro-angiogenic growth factors in high density cultured cells. A, Pathway analysis of microarray data. Genes involved in focal adhesion and ECM-receptor interactions were highly expressed in high density cultured cells compared with those in regular density cultured cells. B, Expression of integrin family genes validated by qRT-PCR analysis. Data are presented as the fold increase of gene expression in high density (HD) cultured cells compared with that in regular density (RD) cultured cells (n = 3). *p<0.05. C, Expression of growth factors validated by qRT-PCR analysis. Data are presented as the fold increase of gene expression in high density cultured cells compared with that in regular density cultured cells. (n = 3). *p<0.05. D, ELISA analysis of VEGF, PDGF, TGF-β, HGF, bFGF and SDF-1α in the supernatants of cultured cells. (n = 3). *p<0.05. E, Tube formation of HUVECs on Matrigel induced by conditioned medium from regular (CM-RD) or high density (CM-HD) cultures. Cells incubated in DMEM with 10% FBS served as a control medium (Ctrl-M). The number of branch points per field was counted after 12 hours of network formation (n = 9). *p<0.05. F, Growth factor expression of CD45^+^ and CD45^−^ cells sorted from high density culture validated by qRT-PCR analysis. (n = 3). *p<0.05.

Global gene expression pattern difference between the high density and the regular density cultured cells was analyzed by a microarray assay. Pathway analysis showed that genes involved in focal adhesion and ECM-receptor interactions were highly expressed in the high density culture (Fig. 6A, Tables S2, S3). In addition, several angiogenesis-related genes were also up-regulated in the high density culture (Table S4). qRT-PCR analysis confirmed that the integrin family (integrin-α1, -α5, -α8 and -α11), their downstream gene FAK, and major pro-angiogenic factors, including VEGF-A, PDGF-B, HGF, bFGF and SDF-1α, were up-regulated in the high density culture (Fig. 6B, C). ELISA analysis of the culture medium showed that higher levels of released VEGF, PDGF, TGF-β and HGF were found in the high density culture than in the regular density culture (Fig. 6D). Interestingly, the conditioned medium derived from high density culture could better induce tube formation of HUVECs on matrigel (Fig. 6E). Further qRT-PCR analysis of sorted CD45^+^ and CD45^−^ cells from HD culture showed that CD45^+^ cells expressed higher levels of VEGF, PDGF, TGF-β and bFGF compared to CD45- cells (Fig. 6F). Collectively, these data demonstrate that the high density culture could potentially enrich EPCs likely via the regulating cell adhesion molecule expression and secretion of growth factors.

## Discussion

In the current study, we established a novel cell culture system in a rat model by seeding bone marrow cells in high density dots. Cells expanded in this culture system displayed an enriched EPC phenotype and a strong therapeutic potential in rescuing ischemic hind limb in vivo.

Since the first discovery of EPCs by Asahara et al. [1], many attempts have been made to expand EPCs in culture, including the pre-coating of culture dishes with ECM proteins and the addition of growth factors to culture medium to mimic the stem cell niche of bone marrow [4], [5], [15]. Nevertheless, the mimicking of cell-cell interaction of the niche environment has remained less thoroughly explored in the expansion of EPCs. The current high density culture system was developed to mimic such a niche factor for better maintaining cell-cell interaction during culture and to avoid the use of additional growth factors. Indeed, with this simple and relatively safe culture method, EPCs could be efficiently expanded and enriched, as the expanded cells exhibited strong expression of EPC related markers (Fig. 2D). More importantly, these cells exhibited angiogenic potential by forming better tubular network in vitro and demonstrating a potent ability to rescue ischemic limbs in vivo (Fig. 4A, B, C). These data provide evidence that this simple culture method can efficiently enrich bone marrow contained EPCs.

As indicated in the published literature [16], [17], cell-matrix interaction and cell-cell interaction among different cell types are considered to be an important part of the mechanism for niche mediated stemness maintenance. As noted, the small cells grew on the top of the spindle-shaped stromal cells (Fig. 2B), probably because of the faster adhesion of stromal cells to the culture plates than the adhesion of other bone marrow cells. We found that only 30 min was required for stromal cells to attach onto culture dishes. Therefore, in high density culture, a fast coating of stromal cells could be achieved rapidly after cell seeding, resulting in the growth of the remaining bone marrow cells on the top of the stromal cells. Apparently, this “cell coating” provided an optimal layer of cells that are able to support the growth of other bone marrow cells on them via a proper cell-cell interaction. Additionally, stromal cell produced matrices are also likely to provide proper cell-matrix interaction that is needed in the native niche environment for maintaining the stemness of EPCs.

In the high density culture, the cell-cell interaction was likely to be mediated via adhesion molecules such as integrin-α1, -α5, -α8 and -α11as their expression was significantly up-regulated. Enriched growth factors favoring the growth of EPCs and their differentiation towards endothelial cell types may also play an important role. As shown in Figure 6C, one to two folds more VEGF, TGF-β, HGF and bFGF were released by high density cultured cells compared to low density cultured cells. The underlying mechanism between cell density and gene expression is still under investigation.

During cell characterization, flow cytometric analyses showed the majority of cells expressing KDR or CD34 also expressed CD45 (Fig. 3C). Culture of isolated CD45^+^ cells confirmed that cells with endothelial-differentiation potential were present inside the CD45^+^ population (Fig. 3D). In addition, co-expression of CD45 and KDR was likely unrelated to cell culture, because co-expression of these markers was observed in freshly isolated bone marrow cells (data not shown). This result coincides with Asahara’s finding that EPCs express CD45 [1]. Recently, Wara et al. demonstrated that common myeloid progenitors and granulocyte macrophage progenitors in bone marrow were able to differentiate into endothelial cells in vitro and in vivo [18]. These findings, in conjunction with the present results, suggest that endothelial precursors might be derived from hematopoietic cells. However, the study from Yoder et al. demonstrated that only endothelial colony forming cells, which do not express CD45, were able to form *de novo* blood vessels in vivo [19], [20]. The relationship between hematopoietic and endothelial precursors remains unclear and worthy of further investigation.

Although EPCs can be enriched by cell sorting based on CD45 expression, cell purification may not be necessary for the purpose of therapeutic in vivo application. It is well known that bone marrow contains several stem/progenitor cell types, including hematopoietic stem cells, mesenchymal stem cells, and EPCs, as well as their descendants [21]–[23]. Actually, a mixed cell population may better promote neovascularization than purified EPCs. Increasing evidence has revealed that mesenchymal cells can support endothelial cells in neovascularization [24], [25]. In addition, injection of bone marrow mesenchymal stem cells was also able to rescue ischemic tissue in several ischemic models by directly differentiating into endothelial cells or by the paracrine actions of growth factors [26], [27]. In this study, several factors may contribute to successful ischemia rescue. One is the differentiation of EPCs to endothelial cells and their integration into newly developed capillary network as donor-derived differentiated endothelial cells were observed in the ischemic area (Fig. 5A, B). Second, the paracrine function of injected cells may also play an important role as both microarray analysis and ELISA demonstrated up-regulated gene expression and protein release of pro-angiogenic factors including VEGF, PDGF and TGF-β (Fig. 6), which can promote neovascularization [28]–[30]. Enhanced tube formation as seen in the in vitro assay using conditioned medium derived from high density culture also provides further evidence supporting the release of growth factors that may support neovascularization.

As a novel EPC culture method, two points need to be mentioned. First, primary culture of whole bone marrow cells is necessary to remove the non-adherent blood cells. Due to the contamination with non-adherent blood cells, we failed to create high density dots by seeding whole bone marrow cells directly on plates without primary culture (data not shown). Second, the number of dots in each plate should be controlled, since increasing the number of dots could quickly reduce the space and exhaust the nutrients for cell expansion. In an additional study, a total of 18 dots were tested in a 10-cm dish. Although no significant change in cell surface marker expression profile was observed compared to those seeded with 6 dots, the medium required daily changes, and cells required passaging every 4 days (unpublished data).

In summary, the current study established a novel and simple cell culture method that expands bone marrow EPCs without additional growth factors. Cells from this culture system may provide a new therapeutic cell source for treating ischemic diseases.

## Supporting Information

Table S1 **Primers used in qRT-PCR analyses.**
(DOC)

Table S2 **Focal adhesion-associated genes with significant up-regulation in high density culture versus regular density culture.**
(DOC)

Table S3 **ECM-associated genes with significant up-regulation in high density culture versus regular density culture.**
(DOC)

Table S4 **Angiogenesis-associated genes with significant up-regulation in high density culture versus regular density culture.**
(DOC)
