# Supplementary material for: Expansion of Endothelial Progenitor Cells in High Density Dot Culture of Rat Bone Marrow Cells
Source: PLoS One. 2014 Sep 25;9(9):e107127. doi: 10.1371/journal.pone.0107127 (PMC4177845; doi:10.1371/journal.pone.0107127)
Supplement: Table S1 — Primers used in qRT-PCR analyses. (DOC) [file pone.0107127.s001.doc]

**Table S1** **Primers used in qRT-PCR analyses**

| **Gene** | **Forward (5’–3’)** | **Reverse (5’–3’)** | **Annealing temperature (°C)** | **Amplified**  **size** |
| --- | --- | --- | --- | --- |
| Integrin-α1 | GTCATCCCTCATAACACCACC | TCCCATCCTCCATCTTGTAG | 86.2 | 178 |
| Integrin-α5 | CGGAGTCCTATTACCCACA | TACAGCCACAGAGTATCCCA | 85.9 | 110 |
| Integrin-α8 | GAGACGCAGACATAGACAAGA | GGGCATCCACTGTTACAACT | 83.2 | 108 |
| Integrin-α11 | CGGCTCCACAGCATCTA | ACGGCATCTTCTCCATACTGA | 85.6 | 123 |
| Integrin-β1 | CATCCCAGCAAGTCCCAAGT | TCTCCCAACACGACCCTCA | 84.1 | 85 |
| Integrin-β3 | GCTGTCCTTCAATGCCACC | GCTCACCGTGTCTCCAATCTT | 85 | 88 |
| FAK | ACAGATGACTATGCCGAGAT | CAATACAGCGTCCAAGTTC | 81.2 | 106 |
| VEGF-A | CCACACCACCATCGTCAC | CCAGAAACAAAACTCCCTAATC | 87.6 | 230 |
| PDGF-B | AGTGTGGGCAGGGTTATT | CGAGGGGAACAACATTATC | 81.1 | 80 |
| TGF-β | GAAGGACCTGGGTTGGAA GT | CGGGTTGTGTTGGTTGTAGAG | 87.5 | 136 |
| HGF | CCTATTTCCCGTTGTGAAG | ACTAACCATCCACCCTACTG | 80.7 | 138 |
| bFGF | CTGCTGGCTTCTAAGTGTG | CAACTGGAGTATTTCCGTG | 80.7 | 98 |
| SDF-1α | AGAGCCAACGTCAAACATCT | CAGGGGTCTACTGGAAAGTC | 83.2 | 186 |
| GAPDH | GTGCCAGCCTCGTCTCATAG | GAACTTGCCGTGGGTAGAGTC | 85.9 | 187 |
