# Supplementary material for: Expansion of Endothelial Progenitor Cells in High Density Dot Culture of Rat Bone Marrow Cells
Source: PLoS One. 2014 Sep 25;9(9):e107127. doi: 10.1371/journal.pone.0107127 (PMC4177845; doi:10.1371/journal.pone.0107127)
Supplement: Table S2 — Focal adhesion-associated genes with significant up-regulation in high density culture versus regular density culture. (DOC) [file pone.0107127.s002.doc]

**Table S2** **Focal adhesion-associated genes with significant up-regulation in high density culture versus regular density culture**

| **Accession number** | **Gene symbol** | **Description** | **Fold change** |
| --- | --- | --- | --- |
| NM_001127449 | *ACTG1* | Actin, gamma 1 (Actg1) | 2.1 |
| NM_031005 | *ACTN1* | Actinin, alpha 1 (Actn1) | 3.1 |
| NM_133651 | *CAV1* | Caveolae protein (Cav1), transcript variant 2 | 2.8 |
| NM_001135009 | *COL4A1* | Collagen, type IV, alpha 1 (Col4a1) | 3.4 |
| NM_134452 | *COL5A1* | Collagen, type V, alpha 1 (Col5a1) | 2.7 |
| NM_021760 | *COL5A3* | Collagen, type V, alpha 3 (Col5a3) | 3.0 |
| NM_001109008 | *COL6A3* | Procollagen, type VI, alpha 3 (Col6a3) | 2.3 |
| NM_001134599 | *FLNA* | Filamin A, alpha (Flna) | 2.8 |
| NM_019306 | *FLT1* | Fms-related tyrosine kinase 1 (Flt1) | 2.8 |
| NM_030994 | *ITGA1* | Integrin, alpha 1 (Itga1) | 4.4 |
| NM_001108156 | *ITGA11* | Integrin, alpha 11 (Itga11) | 2.5 |
| NM_153720 | *ITGB3* | Integrin, beta 3 (Itgb3) | 2.1 |
| NM_001100885 | *MYL9* | Myosin, light chain 9, regulatory (Myl9) | 11.3 |
| NM_001105874 | *MYLk* | Myosin light chain kinase (Mylk) | 23.7 |
| NM_019210 | *PAK3* | P21 protein (Cdc42/Rac)-activated kinase 3 (Pak3) | 2.3 |
| NM_012801 | *PDGFA* | Platelet-derived growth factor alpha polypeptide (Pdgfa) | 2.1 |
| NM_013022 | *ROCK2* | Rho-associated coiled-coil protein kinase 2 (Rock2) | 2.1 |
| NM_001100716 | *SOS1* | Son of sevenless homolog 1 (Drosophila) (Sos1) | 2.4 |
| NM_053861 | *TNC* | Tenascin C (Tnc) | 2.9 |
| NM_001107189 | *TNN* | Tenascin N (Tnn) | 3.6 |
| NM_001107248 | *VCL* | Vinculin (Vcl) | 2.6 |
|  |  |  |  |
