# Supplementary material for: Expansion of Endothelial Progenitor Cells in High Density Dot Culture of Rat Bone Marrow Cells
Source: PLoS One. 2014 Sep 25;9(9):e107127. doi: 10.1371/journal.pone.0107127 (PMC4177845; doi:10.1371/journal.pone.0107127)
Supplement: Table S3 — ECM-associated genes with significant up-regulation in high density culture versus regular density culture. (DOC) [file pone.0107127.s003.doc]

**Table S3 ECM-associated genes with significant up-regulation in high density culture versus regular density culture**

|  |  |  |  | |  |
| --- | --- | --- | --- | --- | --- |
| **Accession number** | **Gene symbol** | **Description** | | **Fold change** | |
| NM_001135009 | *COL4A1* | Collagen, type IV, alpha 1 (Col4a1) | | 3.4 | |
| NM_134452 | *COL5A1* | Collagen, type V, alpha 1 (Col5a1) | | 2.7 | |
| NM_021760 | *COL5A3* | Collagen, type V, alpha 3 (Col5a3) | | 3.0 | |
| NM_001109008 | *COL6A3* | Procollagen, type VI, alpha 3 (Col6a3) | | 2.3 | |
| XM_001060689 | *COL12A1* | Collagen, type XII, alpha 1 (Col12a1) | | 2.5 | |
| NM_198747 | *COL27A1* | Collagen, type XXVII, alpha 1 (Col27a1) | | 4 | |
| NM_001079888 | *MMP28* | Matrix metallopeptidase 28 (Mmp28) | | 3.7 | |
| NM_053606 | *MMP23* | Matrix metallopeptidase 23 (Mmp23) | | 2.6 | |
| NM_012864 | *MMP7* | Matrix metallopeptidase 7 (Mmp7) | | 3.5 | |
| NM_001105925 | *MMP17* | Matrix metallopeptidase 17 (Mmp17) | | 2.6 | |
| NM_022221 | *MMP8* | Matrix metallopeptidase 8 (Mmp8) | | 3.3 | |
| NM_013026 | *SDC1* | Syndecan 1 (Sdc1) | | 4.1 | |
| NM_053893 | *SDC3* | Syndecan 3 (Sdc3) | | 2.6 | |
| NM_012649 | *SDC4* | Syndecan 4 (Sdc4) | | 2.4 | |
|  |  |  | |  | |
