# Supplementary material for: Expansion of Endothelial Progenitor Cells in High Density Dot Culture of Rat Bone Marrow Cells
Source: PLoS One. 2014 Sep 25;9(9):e107127. doi: 10.1371/journal.pone.0107127 (PMC4177845; doi:10.1371/journal.pone.0107127)
Supplement: Table S4 — Angiogenesis-associated genes with significant up-regulation in high density culture versus regular density culture. (DOC) [file pone.0107127.s004.doc]

**Table S4** **Angiogenesis-associated genes with significant up-regulation in high density culture versus regular density culture**

| **Accession number** | **Gene symbol** | **Description** | **Fold change** |
| --- | --- | --- | --- |
| NM_013174 | *TGFB3* | Transforming growth factor, beta 3 (Tgfb3) | 7.5 |
| NM_012620 | *SERPINE1* | Serine peptidase inhibitor, clade E, member 1 (Serpine1) | 5.2 |
| NM_012611 | *NOS2* | Nitric oxide synthase 2, inducible (Nos2) | 4.5 |
| NM_053819 | *TIMP1* | TIMP metallopeptidase inhibitor 1 (Timp1) | 3.1 |
| NM_019147 | *JAG1* | Jagged 1 (Jag1) | 2.6 |
| NM_031511 | *IGF2* | Insulin-like growth factor 2 (Igf2) | 2.5 |
| NM_145098 | *NRP1* | Neuropilin 1 (Nrp1) | 2.2 |
| NM_012886 | *TIMP3* | TIMP metallopeptidase inhibitor 3 (Timp3) | 2.1 |
